# Digital Health Intervention to Increase Health Knowledge Related to Diseases of High Public Health Concern in Iringa, Tanzania: Protocol for a Mixed Methods Study

**DOI:** 10.2196/25128

**Published:** 2021-04-22

**Authors:** Christine Holst, Felix Sukums, Bernard Ngowi, Lien My Diep, Tewodros Aragie Kebede, Josef Noll, Andrea Sylvia Winkler

**Affiliations:** 1 Centre for Global Health, Department of Community Medicine and Global Health Institute of Health and Society University of Oslo Oslo Norway; 2 Directorate of ICT Muhimbili University of Health and Allied Sciences Dar es Salaam United Republic of Tanzania; 3 Muhimbili Medical Research Centre National Institute for Medical Research Dar es Salaam United Republic of Tanzania; 4 Mbeya College of Health and Allied Sciences University of Dar es Salaam Mbeya United Republic of Tanzania; 5 Oslo Centre for Biostatistics & Epidemiology Oslo University Hospital Oslo Norway; 6 Fafo Institute for Labour and Social Research Oslo Norway; 7 Basic Internet Foundation Kjeller Norway; 8 Department of Technology Systems University of Oslo Oslo Norway; 9 Centre for Global Health, Institute of Health and Society University of Oslo Oslo Norway; 10 Center for Global Health, Department of Neurology Technical University of Munich Munich Germany

**Keywords:** digital health, eHealth, mHealth, DigI, Tanzania, digital health messages, digital health promotion, digital health education, HIV/AIDS, tuberculosis

## Abstract

**Background:**

Traditionally, health promotion and health education have been provided to communities in the global south in the form of leaflets or orally by health care workers. Digital health interventions (DHIs) such as digital health messages accessed by smartphones have the potential to reach more people at a lower cost and to contribute to strengthening of health care systems. The DHI in this study focuses on disseminating digital health education regarding 3 disease complexes of high public health concern: HIV/AIDS, tuberculosis, and *Taenia solium* (neuro)cysticercosis or taeniasis, a parasitic zoonotic disease that requires a One Health approach. The DHI presents the participants with animated health videos (animations) and provides access to information spots (InfoSpots) with a free-of-charge digital health platform containing messages about health to rural Tanzanian communities.

**Objective:**

The objective of this study is to measure the effect of the DHI on health knowledge uptake and retention over time in the rural communities.

**Methods:**

This is a mixed methods study including a nonrandomized controlled trial and qualitative interviews conducted in rural Tanzania. A health platform containing digital health messages for the communities was developed prior to the study. The health messages consist of text, pictures, quizzes, and animations of everyday stories, aimed at disease prevention and early treatment. The baseline and immediate postintervention assessments were completed in Iringa, Tanzania in May 2019. The participants were interviewed by enumerators and completed questionnaires regarding health knowledge. Participants in the intervention group were exposed to 3 different health animations once on a tablet device. The participants’ health knowledge was assessed again immediately after the exposure. The first follow-up survey was undertaken in August 2019. The InfoSpots with the digital health platform were thereafter launched in the intervention villages in November 2019. Qualitative interviews were undertaken in February 2020. The second follow-up was completed in June 2020.

**Results:**

A total of 600 participants have been enrolled in the trial. We will assess (1) the difference in knowledge scores between baseline and the immediate postintervention assessments in the intervention group and (2) the difference in knowledge scores between the intervention and control groups at baseline, 3 and 6 months post-DHI rollout. Since a randomized design did not prove feasible, potential confounders (eg, age, gender, education, and time of exposure) may be introduced, and results will be adjusted. Data analysis for the 35 qualitative interviews is currently ongoing, and perspectives and experiences related to use and nonuse of the InfoSpots are being explored.

**Conclusions:**

The data have been collected, and the analysis is ongoing in this digital health study, aimed at evaluating the effects of a DHI based on relevant health messages. The publications of results can be expected this year.

**Trial Registration:**

ClinicalTrials.gov NCT03808597; https://clinicaltrials.gov/ct2/show/NCT03808597

**International Registered Report Identifier (IRRID):**

RR1-10.2196/25128

## Introduction

### Background

Health education is seen as an essential component in efforts to prevent diseases [1]; furthermore, it is the part of health care that is concerned with promotion of healthy behavior [2]. Health education and health promotion are two terms that are sometimes used interchangeably [3]. Health promotion has a wider meaning, defined by the Ottawa charter in 1986 as “the process of enabling people to increase control over, and to improve, their health” [4]. The World Health Organization defines health literacy as something that “represents the cognitive and social skills which determine the motivation and ability of individuals to gain access to, understand and use information in ways which promote and maintain good health” [5]. Health literacy is a key determinant of health; limited health literacy is an underestimated problem and is associated with risky behavior, poorer health, less self-management, and more hospitalization and health care costs [6]. Health literacy is often perceived as the outcome of health education [7].

Increased health literacy levels have been demonstrated, among others, after basic education among adults [8], tailored rehabilitation education among patients who underwent a breast operation [9], education through active learning among elders in rural areas [10], and health belief model–based health education among patients with hypertension [11]. However, additional studies are needed to provide evidence on the best context-specific approach to improve impact on health literacy, health outcomes, and ultimately health disparities.

Strategies to increase understanding and individual health literacy can include delivering only a few health messages at a time, using plain and jargon-free language, resorting to visual aids, and using the teach-back method for health care workers when talking to patients [12].

Creating awareness of the prevalence, causes and transmission, signs and symptoms, and possibilities of treatment and prevention of a disease aims to provide early treatment and increase appropriate use of health care services. This is important in order to reduce the burden of infectious diseases like HIV/AIDS, tuberculosis (TB), and *Taenia solium* (neuro)cysticercosis or taeniasis (TSCT) in rural areas in the global south, where access to health care services may be starkly limited. Traditionally, health education has been the task of health workers or community health workers in such areas.

Over two-thirds (25.7 million) of all people living with HIV live in regions of Africa [13]. Young women between the ages of 15 and 24 years are twice as likely to be living with HIV as men, and in sub-Saharan Africa, girls account for 5 in 6 new infections among adolescents aged 15 to 19 years [14]. Complete and reliable information, rapid diagnosis, and early treatment and care are essential to control the virus, alleviate disease symptoms, and prevent transmission. TB treatment saved over 60 million lives globally between 2000 and 2020. However, despite TB being both treatable and curable, 1.4 million people died from the disease in 2019 [15]. The most efficient way to save lives, apart from prevention, is to diagnose and treat TB as early as possible. TSCT is an emerging but neglected parasitic and zoonotic disease (ie, both humans and animals are infected) caused by the tapeworm *T.* *solium*. In humans, the disease manifests mainly as neurocysticercosis (ie, the settlement of tapeworm larvae in the brain), the most frequent preventable cause of epilepsy in *T. solium* endemic areas of sub-Saharan Africa [16]. TSCT, endemic in Tanzania [17], has been listed as a priority disease for international attention towards elimination and is part of a wider aim to alleviate poverty in the same regions [18]. Iringa in southern Tanzania is endemic also for the other 2 diseases covered in this study, with one of the highest HIV prevalence estimates in the country in 2016 [19] and a notification rate for TB above the national average in 2018 [20].

Today’s education channels are changing. Therefore, health education interventions must meet the target population at respective levels of access and technology [21] and rely on the characteristics of the technology available [22]. Digital health interventions (DHI) representing “a discrete functionality of the digital technology to achieve health sector objectives” [23] and electronic health (eHealth), defined as the use of information and communication technologies for health [24], are now understood as pivotal in order to provide better care to a larger number of people, especially those most in need [25]. eHealth can be regarded as a tool to provide the public with health messages and support as well as to motivate *clients* to care about health. According to the World Health Organization [23], clients are “members of the public that are potential or current users of health services, including promotion activities,” the latter playing a vital role in their health management and disease prevention decisions. eHealth includes mobile health, defined as the use of mobile wireless technologies for health [26], a useful tool to deliver education and promote health-seeking behavior and health-related lifestyle decisions, since people nowadays can be contacted more easily through their phones [27].

Within the Sustainable Development Goals endorsed by the United Nations in 2015, SGD 3, which is about ensuring healthy lives and promoting well-being for all at all ages [28], will not be achieved without giving people access to the information they need in order to live healthier lives. The use of digital tools and technology is also emphasized as a strategy for improving health literacy [29]. However, access to digital information is not enough; in order to utilize digital health resources, a basic digital literacy skill set is needed [30].

The use of smart devices and mobile data services is taken up rapidly, and it is likely that a larger part of the population in, for example, sub-Saharan Africa will have access to both of these in the near future [31]. The Tanzania Communications Regulatory Authority regularly produces reports of mobile service penetration including internet services. The reports do not provide distribution of the services by region or district. The smartphone penetration in Iringa is unknown, but in 2016, 25.3% of national mobile connections were smartphones [32]. The cost of smartphones is a critical barrier to mobile internet adoption in sub-Saharan Africa, especially among people living in rural areas [33], leaving reasons to believe that less than a quarter of the population in rural Iringa have access to other health education than via health care workers at dispensaries with various opening hours.

The Non-discriminating Access for Digital Inclusion project (hereby referred to as the DigI project) addressed this gap by providing access to free digital health messages [34] related to HIV, TB, and TSCT in 2 rural villages in Iringa, Tanzania. Eleven partners from 8 countries [35] are collaborating in the project, including local and international researchers with extensive experience within the aforementioned diseases. The multi- and interdisciplinary approach used in the development and digitization of the health messages referred to in this protocol are further described in a recent paper [36]. In the DigI project, a key performance indicator framework was developed [37], and it is assumed that the digital health intervention described in this protocol will increase digital literacy in addition to health knowledge.

### Objectives

The overarching objective of this study is to assess the effect of the DHI described in the following paragraphs aiming to improve health knowledge in the rural communities of Iringa district, Tanzania. The results will contribute to the body of evidence on the impact of digital health education and promotion in a rural setting in the global south.

The effect of the DHI will be assessed by (1) the health knowledge uptake, comparing baseline assessment (T0) scores and immediate postassessment (T1) scores of the intervention group, after exposure to 3 animations (the first part of the intervention, hereby referred to as Part 1); (2) health knowledge retention, comparing baseline assessment (T0) scores with the first 3-month follow-up (T2) scores after exposure to Part 1 and with the final follow-up (T4) scores of the intervention group, 6 months post InfoSpot rollout (the second part of the intervention, hereby referred to as Part 2); (3) a comparison between the intervention and control groups regarding the changes from T0 to T2 and T4 after participation in Part 1 and Part 2; and (4) an exploration of the perspectives and experiences of the DHI among local users and nonusers using semistructured interviews (T3), after participation in Part 1 and Part 2.

## Methods

This study deploys mixed methods, including a quantitative survey in a nonrandomized controlled trial, in addition to qualitative semistructured interviews.

### Location

The study is conducted in the Iringa district, selected specifically because the prevalences of all 3 diseases are high and it is a rural district in which many villages do not have access to the internet. Migoli and Izazi villages were chosen as intervention villages at an early stage by the DigI team because they are easily accessible from the highway between Iringa town and Dodoma. Kimande and Idodi were chosen as control villages because they are comparable with regards to geographic, demographic, and socioeconomic features to the intervention villages and deemed to be away far enough to keep knowledge contamination by the intervention villages to a minimum. The closest intervention village (Izazi) is located about 32 km from the closest control village (Kimande). However, the road between the 2 villages spans 124 km.

See Table 1 for detailed information on the activities undertaken at the study sites, from April 2019 to June 2020. The health knowledge, health literacy, and digital literacy questions in the questionnaires are identical in T0, T1, T2, and T4.

**Table 1:** Activities at the study sites at each time point.

Activities		April 2019 - May 2019				August 2019		November 2019		February 2020		June 2020
		T0^a^	Part 1^b^	T1^c^	T2^d^		Part 2^e^		T3^f^		T4^d^	
**Intervention group**												
	Group involved in assessments?	Yes	Yes (supervised)	Yes	Yes		Yes (voluntarily, unsupervised)		Yes		Yes	
	Assessments	40 HKQ^g^, 6 HLQ^h^, 5 DLQ^i^	N/A^j^	40 HKQ	40 HKQ, 6 HLQ, 5 DLQ		N/A		Semistructured interviews		40 HKQ, 6 HLQ, 5 DLQ, 9 UIH^k^	
	Sample size, n	300	300	300	300 (minus any dropouts)		300 (minus any dropouts)		35		300 (minus any dropouts)	
**Control group**												
	Group involved in assessments?	Yes	No	No	Yes		No		No		Yes	
	Assessments	40 HKQ, 6 HLQ, 5 DLQ	N/A	N/A	40 HKQ, 6 HLQ, 5 DLQ		N/A		N/A		40 HKQ, 6 HLQ, 5 DLQ	
	Sample size, n	300	N/A	N/A	300 (minus any dropouts)		N/A		N/A		300 (minus any dropouts)	

^a^Baseline assessment, which includes baseline data collection in both groups.

^b^Immediately after T0, the participants in the intervention group are exposed to 3 animations.

^c^Postassessment immediately following Part 1.

^d^T2 and T4 are the follow-up assessments.

^e^Rollout of the second part of the intervention (InfoSpots).

^f^Qualitative interviews.

^g^HKQ: health knowledge questions.

^h^HLQ: health literacy questions.

^i^DLQ: digital literacy questions.

^j^N/A: not applicable.

^k^UIH: use of the InfoSpots and digital health platform.

### Description of the Digital Health Intervention

The intervention described in this protocol is composed of 2 components or parts.

#### Part 1

In April and May 2019, the participants had supervised exposure to 3 animated health videos (hereby referred to as animations).

The participants in the intervention group received the first part of the intervention immediately after the baseline questionnaire. They were exposed to 3 health animations on a 7-inch tablet. These animations contain health messages on (1) HIV/AIDS, (2) TB, and (3) TSCT in the local language (Kiswahili). The health messages explain aspects related to disease prevalence, causes and transmission, signs and symptoms, as well as treatment and prevention. The animations are between 3 minutes and 7 minutes long. They tell personal stories of ordinary people in the community. The stories address the particular disease and show how a person becomes infected, develops signs and symptoms, and can get medical care and how spreading of the disease can be prevented within the communities. The HIV/AIDS animation depicts the story of a woman, recently married, who discovers that she is infected with the virus. The story illustrates how one can get infected by a person one knows and trusts, but also how one can live a fairly normal life with the right medical treatment. The TB animation presents the story of a farmer who gets diagnosed with TB after showing symptoms of TB disease. The animation emphasizes disease prevention efforts covering individual, family, and community levels. The third animation tells the story of a grandfather who gets sick after consuming undercooked pork. The animation focuses on hygiene, hygienic animal handling, and medical treatment if TSCT symptoms occur. All animations can be found online, in both English and Kiswahili [34] (the English versions can be viewed in Multimedia Appendices 1-3). The health knowledge questions from the baseline questionnaire were immediately repeated after the participants in the intervention group were exposed to the animations. Subsequent to the last question, the enumerators explained where and when the information spots (InfoSpots) were planned to be installed and that the participants could access the health messages and internet for free in these local InfoSpots.

#### Part 2

In November 2019, we provided unsupervised community access to InfoSpots providing free health education in the form of text, pictures, quizzes, and animations.

The InfoSpots containing the digital health platform (Multimedia Appendix 4) and providing intervention villages with free access to digital health messages related to the aforementioned diseases were implemented in Tanzania in November 2019. The digital health platform is now accessible free-of-charge, either through people’s own devices, such as smartphones, laptop computers, or tablet devices, or through available community tablets in the InfoSpots, provided by the DigI project to offer access for people without their own devices. In Izazi and Migoli, InfoSpots are located in the village offices, at the local dispensaries, and at the local high school (only in Migoli). The InfoSpots are available for all clients in the intervention villages, not only for the participants in the study described in this protocol.

When clients access the digital health platform, they may choose an area of interest, for example TSCT. Here, they are able to read key messages in all areas: prevalence, causes and transmission, signs and symptoms, treatment and prevention. The key messages are illustrated with snapshots from the animations. Clients are able to answer quizzes, and the correct answers are revealed at the end of each quiz. In addition to text, there are animations for each of the 3 diseases, which can be streamed over a local server. Health messages related to other diseases are also available.

### Study Design

Randomization of subvillages, households (HH), or participants to either the intervention or control group within the villages was not possible, as the InfoSpots are available for all people in the intervention villages and the risk of contamination (knowledge transfer) between participants in the 2 groups would have been too pronounced. This resulted in choosing a nonrandomized design. However, a random sample of participants was selected from the villages as described in the following paragraphs.

To complement the quantitative findings, semistructured interviews with participants in the intervention villages were included to explore experiences and perspectives related to the intervention.

#### Study Population

In Tanzania, 38 million of the 58 million people (65.5% of the population) live in rural areas [38]. Many of the communities are hard to access due to the poorly developed infrastructure. Iringa district, with a projected population of 254,032 in 2019 [39], is located in the southern Highlands of Tanzania bordering the Mbeya, Njombe, Morogoro, Dodoma, and Singida regions. About three-quarters of the adult population are able to read, and agricultural activities play the most important roles in employment in rural Iringa, generating close to the total gross domestic product [40]. In a study from 2017, 6 of 10 adults in Iringa were likely to have at least a primary school education [41]. Almost three-quarters of households in Iringa have earthen floors, and half of the houses have mud walls [40]. One-quarter of the households are female headed, mainly due to the HIV/AIDS epidemic [42].

The latest population census in Tanzania was in 2012 [43], with a total population of 5281 in Izazi, 10,937 in Migoli, 10,202 in Idodi, and 14,420 in Itunundu, the area that covers Kimande. The total populations in the study villages were much lower than depicted in the census according to the consulted village executive officers: 6233 people in Migoli and 1839 people in Izazi. In the control villages, the village executive officers reported the populations to be 3562 in Kimande and 3601 in Idodi.

#### Participants and Recruitment

**Figure 1:**
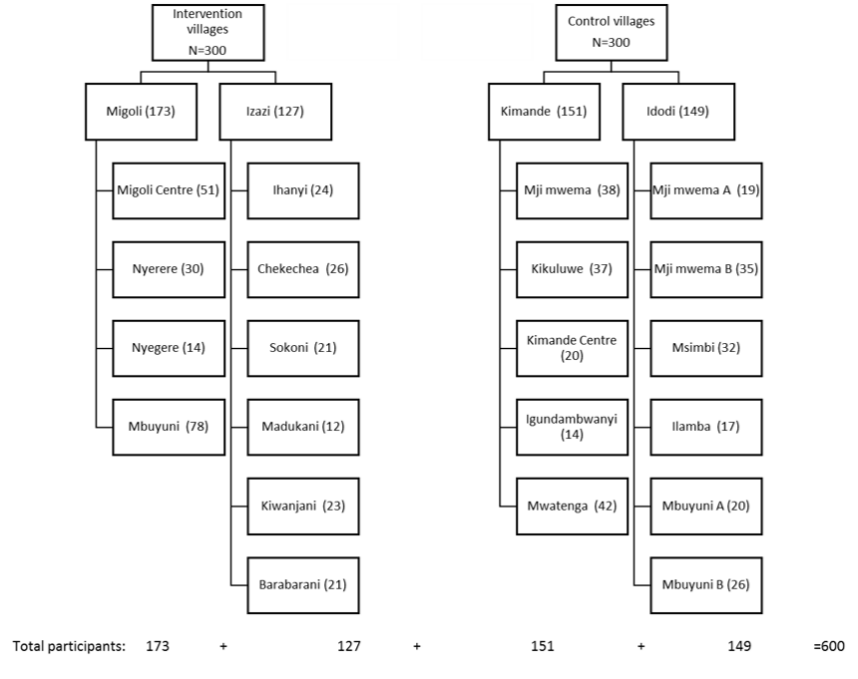
Participants from the respective groups, villages, and subvillages.

Participants were recruited from subvillage lists during 2 phases. The early phase took place in April 2019. Lists of HHs were constructed at the village level based on the village register of inhabitants. In all 4 project villages, migration due to work opportunities is part of the population dynamics. We experienced that the initial lists, created at the village level, consisted of numerous HHs that had already moved to other places and that some of the randomly selected HHs were absent. Consequently, additional lists were created in May 2019, achieved by the subvillage chairpersons going door to door in their respective communities to complete and update the list of the subvillage HHs. The HHs with participants that were already enrolled in the study were removed from the updated subvillage lists but kept in the study, and additional HHs were selected to meet the required sample size. These 2 lists are the foundation of the selected HHs from each subvillage, according to probability proportionate to size [44]. Please see Multimedia Appendix 5 for the sampling plan containing calculations of the proportions to be selected from each subvillage and the flow diagram of participants from each village and subvillage in Figure 1.

The sampling unit in this study is the HH, randomly selected by a random number generator from the subvillage lists. We used the Kish grid [45], which is a sampling method using a preassigned table of random numbers when selecting the one participant per HH [46].

During the process, the subvillage chairpersons guided each of the 6 enumerators to each of the selected HHs in their respective subvillages. All communication between the research team and the participant was conducted in the local language. The enumerators were properly introduced by the village chairpersons, and the HHs were reassured of the trustworthiness of the research project by the chairpersons. Following a brief presentation of the study by the enumerator, the approached HH member, normally the head of the HH, sat with the enumerator and listed all HH members so that the enumerator could randomly select a participant from the HH via the Kish selection method.

The inclusion criteria are (1) aged 15-45 years; (2) permanently living in the randomly selected HH or, at least, planning to not move during the next 8 months; (3) being capable and willing to sign an informed consent form or, in the case of illiterate participants, to sign with their thumbprint.

The exclusion criteria are (1) aged >45 years or <15 years; (2) planning to be absent from the village for more than 3 weeks in the upcoming 8 months; (3) not being able or willing to give written informed consent or, in the case of illiterate participants, a thumbprint.

The participants in the intervention group were assessed for baseline health knowledge (T0) related to the intervention diseases in Migoli and Izazi (the intervention villages) before being exposed to the 3 animations. The latter represent the first part of the DHI, Part 1, or “description of the intervention.” Consequently, the participants of the same group were given the same questions as in the baseline questionnaire. We call this the immediate post assessment (T1). T0, Part 1 of the DHI, and T1 all took place on the same day for the each of the participants in the intervention group. The control group consists of another 300 individuals in the control villages Idodi and Kimande. In the control villages, the participants were assessed for their baseline health knowledge (T0) in the same way as in the intervention group and were given the same questionnaire.

The participants in both groups were followed up in T2 with the same questions as in T0. When the data collection from T2 was completed, village InfoSpots with access to the digital health platform with the health messages were launched in the intervention villages. This represents the second part of the DHI: Part 2. Follow-up surveys with the same questions as in T0 were repeated in both groups 6 months after the rollout of Part 2 (T4), in June 2020.

The participants in the qualitative component were randomly selected from the list of participants enrolled in the quantitative study or were recruited on site as users of the InfoSpots.

#### Outcomes

The intervention period lasted from April 2019 until June 2020. Primary outcomes of this study are focusing on health knowledge retention in the intervention group compared to the control group, 6 months after Part 2, which is 12 months after Part 1. Secondary outcomes are focusing on immediate health knowledge uptake in the intervention group only, as a result of the exposure to the animations in Part 1.

#### Ethics and Informed Consent

This protocol is registered in Helseforsk, University of Oslo, Norway, and the project has been assessed by the Norwegian Centre for Research Data (NSD), reference number 59643. Ethical approval from the National Institute for Medical Research in Tanzania has been granted twice with the reference number NIMR/HQ/R.8a/Vol IX/2947. The study is registered in ClinicalTrials.gov with ID: NCT03808597. Additionally, the first author was provided with a research permit from the Tanzanian Commission for Science and Technology.

All participants were briefed about the aims of the study, their voluntary participation, and their rights as participants according to The European Union General Data Protection Regulation (GDPR). The participants were informed about the types of personal data collected (name, sex, age, education, religion, mobile number), where the data was going to be kept, and for how long and who would have access to these data. In addition to this oral briefing, participants were given a letter containing the information, along with the contact information of the local research entity. Informed consent forms were signed by all participants willing to participate in the study and by one enumerator. The forms were developed with support from NSD.

We are fully committed to good ethical and legal practice. All information about the participants will be dealt with confidentially and will remain deidentified for 2 years after the end of the entire project. The deidentification code and the collected sensitive data will be stored separately in the University of Oslo’s Services for Sensitive Data (TSD) [47]. In 2022, the deidentification code will be deleted, and the material will remain anonymous for public reuse. A possible benefit for the participants taking part in this study is an increase in knowledge of the highly prevalent diseases of HIV/AIDS, TB, and TSCT. The InfoSpots with the digital health platform will continue to be available after the intervention period is over. No intervention was given to the control group, but when data collection ended in June 2020, the InfoSpots with the same digital health platform were implemented in the control villages.

This study focuses solely on the participants’ health knowledge, not on the participants’ own health status or personal health data. The study participants were not given any compensation for taking part in the study.

### Data Collection

The data collection consists of a quantitative part, spanning from April 2019 to June 2020, and a qualitative part, conducted in February 2020.

#### Collection of Quantitative Data Through Questionnaires

The questionnaire (Multimedia Appendix 6) used in T0, T1, T2, and T4 contains health knowledge questions concerning all 3 diseases, covering the domains of prevalence, causes and transmission, signs and symptoms, as well as treatment and prevention. The prevalence questions explored whether the participants had heard about the diseases. In the causes and transmission domain, we questioned the participants about how the disease transmits or what the cause of the infection is. The participants were asked to describe signs and symptoms for the relevant disease and how the diseases are diagnosed. Further, we asked questions related to treatment and cure. Lastly, the participant answered questions regarding different prevention strategies, both for individuals and communities. The questions can have one or several correct answers. The enumerators read the questions to the participants but did not inform people about the possible answers. All questions are thus open-ended. The questionnaire contains questions accompanied by a list of possible answers, so the enumerator can check the answer provided by the participant.

We used 13 questions in the HIV/AIDS section: 1 related to prevalence, 4 related to causes and transmission, 2 related to signs and symptoms, 3 related to treatment, and 3 related to prevention. We also asked the participants where they could find sufficient and reliable health information on HIV/AIDS in their communities. In the next section, the participants answered 10 questions related to TB: 1 in the prevalence domain, 2 in the causes and transmission domain, 2 related to the signs and symptoms, 3 related to treatment and, finally, 2 regarding prevention. The last section was comprised of 17 questions related to TSCT. Since this is a relatively unknown disease, we came up with 4 questions related to the prevalence of the disease, in the case of both pork tapeworm and cysticercosis. We used 4 questions in the causes and transmission domain, 4 questions in the signs and symptom domain, 1 question related to treatment, and 4 questions concerning prevention. The participants also answered 6 health literacy questions, in addition to 5 digital literacy questions.

During T4, we asked the participants of the intervention group whether they had taken advantage of the InfoSpots and the digital health platform and which part of the platform they had accessed, before repeating the questionnaire.

The study questionnaire, containing health knowledge questions regarding our 3 diseases of interest, digital and health literacy, and the use of the InfoSpots, was developed within the DigI group over a 6-month period in 2018. We used validated questions from earlier studies [48] and reports (TACAIDS/Ministry of Home Affairs, 2012; International Organisation for Migration, 2014) from various collaborations with DigI team members, as much as possible.

The DigI group had developed the key messages we wanted to convey in the health animations [36] from reliable and approved health education sources. Some of the questions were derived from this work. One key message within the HIV section, for example that girls and young women are the most vulnerable group, was emphasized in the HIV animation, and the question derived from this key message simply reads, “Which group do you think is most vulnerable to HIV infection?”

When collecting data from the questionnaire, an Android App called KoBoCollect [49] was used. The questionnaire was uploaded to KoBoToolbox [50], software for conducting field data collection in challenging environments. This is a free, open-source tool that allows users to create and deploy questionnaires to be used in an area with no internet connection.

Accessing KoBoToolbox is only possible with a username and password. The questionnaire was shared with the enumerators, who were able to submit data but could not access the collected information. Once the tablets were connected to Wi-Fi or mobile internet, the app automatically transferred the collected data to the online platform KoBoToolbox.

During the first survey round, the participants were given questions concerning their demographic information. This personal information — name, mobile phone number, and village — was registered on the HH list but not in KoBoCollect. Neither was it uploaded to KoBoToolbox, so to reduce the risk of data privacy violation. A digital object identifier (DOI) was created for each participant, and the DOI was registered in KoBoCollect along with age and sex. Thus, no personal information was uploaded to KoBoToolbox. The personal identification key was stored directly in TSD via the tablets and not transmitted via email.

The reason for asking the participants for their names and mobile phone numbers was to be able to relocate the participants in the follow-up surveys. During the follow-ups in T2 and T4, the research staff had lists with names and DOIs. When entering the data from the follow-ups, the enumerators only had to identify the participants according to the list and to use the DOI when collecting new data in KoBoCollect.

#### Collection of Qualitative Data Through Semistructured Interviews

In order to complement the quantitative data, 35 semistructured interviews with people from the intervention villages were conducted at T3 (ie, 3 months after Part 2). The main aim was to understand the respondents’ perspectives on the intervention [51]. Results from this data collection will provide in-depth information on how the intervention was perceived and received in the communities. It may also shed light on the possible effects of the intervention, raise opportunities and problems, and provide a different view on the use of the InfoSpots along with the digital health messages.

An interview guide (Multimedia Appendix 7) was prepared in order to steer the conversations and to make sure that no question remained unanswered or unclear. The interviews were conducted between clients in the intervention villages and the first author of this paper, in collaboration with a Tanzanian Kiswahili translator. The participants were picked randomly from the same lists as in the quantitative study and by convenience sampling in and around the InfoSpots, to include users not associated with the quantitative study. The semistructured interviews were recorded with a recording app from the University of Oslo and transcribed afterwards. The records and transcriptions were stored separately from the deidentification code, in TSD, to ensure anonymity and fulfil the data handling requirements of the GDPR. The deidentification code will be deleted 2 years after the project is completed. Afterwards, the data will remain anonymous in the NSD archives.

#### Training and Piloting of the Questionnaire

A 3-day training for the enumerators was held in Morogoro, Tanzania, in March 2019; 6 enumerators took part. The enumerators were familiarized with the tablets and the questionnaire installed on the tablets. They practiced the registration in TSD and learned how to provide the participant with the relevant information and contact details, in addition to obtaining informed consent. Mock interviews, using the tablets with a training version of the questionnaire, followed by submission of questionnaires via KoBoCollect, were staged.

The team went to the piloting site, Luhindo primary school, located about 40 minutes from Morogoro, on the second day. Partners from Sokoine University of Agriculture had arranged to pilot the questionnaire with a group of people working and living in the Morogoro area. Altogether, 50 submissions were entered into KoBoCollect.

On the final day of training, the Kish selection method was practiced. Registration of all participants from the pilot into TSD the day prior was performed, assuring that the personal data were stored carefully in TSD whilst their anonymous answers to the disease knowledge, digital literacy, and health literacy questions were transferred to KoBoToolbox.

All the posed questions were reviewed by the pilot participants. Seven questions were altered, and some alternative responses were added.

Prior to the training, the study tools had been translated from English to Swahili. After the training, the questionnaire was revised in the local language and translated back to English. This procedure was also applied to the consent forms and information letter.

### Data Analysis Plan

#### Analysis of Quantitative Data

We will assess the effect of the DHI by calculating the number of correct answers and the knowledge scores in both groups at all set time points. The first calculation will be related to the baseline assessment (T0) in the intervention and control group, the immediate postassessment (T1) in the intervention group, followed by the number of correct answers and scores at 3 months (T2) post Part 1 and 6 months (T4) post Part 2, in both groups. The scores will be calculated with 1 point for each correct response and summarized for each disease and the various domains (eg, causes and transmission or signs and symptoms). The number of correct answers and scores will be applied when comparing the 2 groups and the same group at different time points. The number of correct answers and the scores will be used to follow individual participants over time, as well as to indicate the trends in the increase in health knowledge uptake and retention at community level.

A sample size of approximately 460 participants was required to detect a difference of 15% to 20% between 2 groups with and without intervention, assuming that the proportion of correct responses was 50% in the group without intervention. A 2-sided significance level of .016 and 80% power were used. The significance level was set at .016 (using Bonferroni correction) to compensate for 3 time points since it was not known which time point would become relevant for the comparison between the groups. We decided to include 600 participants to increase precision of the key estimates and take dropouts into account.

The data will be analyzed in Stata/SE version 16. The McNemar test for paired data will be applied to determine marginal homogeneity of 2 dichotomous variables. The scores will be assessed against other variables such as age, education level, and gender. Summary tables with calculated averages (mean, median, and mode) will be created. We will compare between the groups at baseline, 3 and 12 months, with and without adjusting for variables that could have influenced the respective changes. Mixed-effect models will be used to estimate the difference and monitor the alterations of variables for each question, domain score, and overall score.

#### Analysis of Qualitative Data

The data from the interviews will be analyzed in the software NVivo. A coding frame will be developed, and a content analysis method will be applied [52]. Themes of interest from the interview guide, as well as new themes found in the data, will be analyzed to contextualize the participants’ perspectives and experiences. When analyzing and summarizing the interviews, a table will be developed with excerpts from the most important findings of the qualitative research, alongside quotes from the interviews.

## Results

The data collection was completed recently, and no results can be presented at this stage. Recruitment started April 1, 2019 and ended May 25, 2019. The 600 participants recruited have been followed over 1 year. However, 106 participants dropped out before T4, leaving 494 participants for complete follow-up analysis. The InfoSpots were launched in the intervention villages in November 2019 and in the control villages in June 2020. By enabling free-of-charge use of the InfoSpots for all community members, the approach is inclusive and not only focused on empirical findings.

The 35 qualitative interviews were undertaken in February 2020. All data are currently being analyzed, and the results are expected to be published in 2021.

## Discussion

The primary aim of this study is to evaluate the effect of the DHI on the rural population in Iringa, Tanzania.

The quantitative part will provide a broad, “hard scientific” overview of the trends in correct answers (ie, knowledge scores) over time within and between intervention and control groups. By counting the number of correct answers before and after the intervention and comparing the knowledge scores of the respective groups, we will be able to see whether the intervention has successfully increased health knowledge in the intervention group.

As the quantitative approach only focuses on numbers, not the people themselves, the team decided to also include a qualitative component. Semistructured interviews will allow the researchers to apply an informal tone with the participants, and the findings will add meanings, opinions, values, feelings, and personal experiences to the quantitative research. The interviews can also function as arenas for discussion and may contribute to the validation of the quantitative findings.

The use of mixed methods will provide a well-founded understanding of the project results [53]. Combining the quantitative with the qualitative findings will lead to a robust set of data, not just to evaluate the DHI but also as a basis for adjusting the intervention regarding implementation of both the digital health messages and the InfoSpots with access to the digital health platform. Drawing upon findings from both research approaches to improve the intervention, the project demonstrates a participatory and community-based component that has the potential to lead to a better, context-specific impact on digital health education of local communities.

### Limitations

This study has several limitations. The results may not be applicable to the greater rural population in Tanzania, since only a small number of villages and subvillages were included in the study. Although the study accounted for anticipated dropouts by including 100 additional participants in the study sample, this is an environment in which people shift location frequently. The rate of dropouts may be larger than anticipated in T2 and T4. People in the communities migrate as a result of the season, rainfall, drought, or harvest. A high dropout rate may introduce bias. Dropout analysis will be conducted.

Another limitation is that the participants in the intervention group may have influenced other potential participants of the same group. Data collection for T0 and T1 in the intervention villages was ongoing for 2 months. It is possible that some participants in the intervention group may have contaminated the findings by talking about the health animations they were exposed to in Part 1. Also, the research team cannot control the participants from the intervention group, who might travel to the control villages and inform recruited participants about the DHI.

### Conclusions

We anticipate that our study will contribute to increasing knowledge of HIV/AIDS, TB, and TSCT as well as increasing overall health and digital literacy for individuals and communities in Iringa, Tanzania. By providing health messages in a digital format, the participants and their immediate surroundings will increase their health-related knowledge, which has potential to lead to an adaptation in health-seeking behavior, although this was not investigated in our study. Digitization of health information aimed at people in rural areas of sub-Saharan Africa may also contribute to strengthening of health systems, especially in low-income settings.

## Appendix

Multimedia Appendix 1 “The story of tapeworms” - TSCT animation.

Multimedia Appendix 2 “The story of HIV” - HIV animation.

Multimedia Appendix 3 “The story of TB” - TB animation.

Multimedia Appendix 4 Screenshot of the digital health platform - health information.

Multimedia Appendix 5 Sampling plan.

Multimedia Appendix 6 Study questionnaire (HIV / TB / TSTC knowledge questions used at T0/T1, T2 and T4).

Multimedia Appendix 7 Interview guide for qualitative study.

## Abbreviations

DHI: digital health intervention
DigI: Non-discriminating Access for Digital Inclusion
DOI: digital object identifier
eHealth: electronic health
GDPR: The EU General Data Protection Regulation HH: Household
NSD: Norwegian Centre for Research Data
TB: tuberculosis
TSCT: *Taenia solium* (neuro)cysticercosis or taeniasis
TSD: Services for Sensitive Data (at University of Oslo)
