# Supplementary figures and images for: Digital Health Intervention to Increase Health Knowledge Related to Diseases of High Public Health Concern in Iringa, Tanzania: Protocol for a Mixed Methods Study
Source: JMIR Res Protoc. 2021 Apr 22;10(4):e25128. doi: 10.2196/25128 (PMC8103301; doi:10.2196/25128)

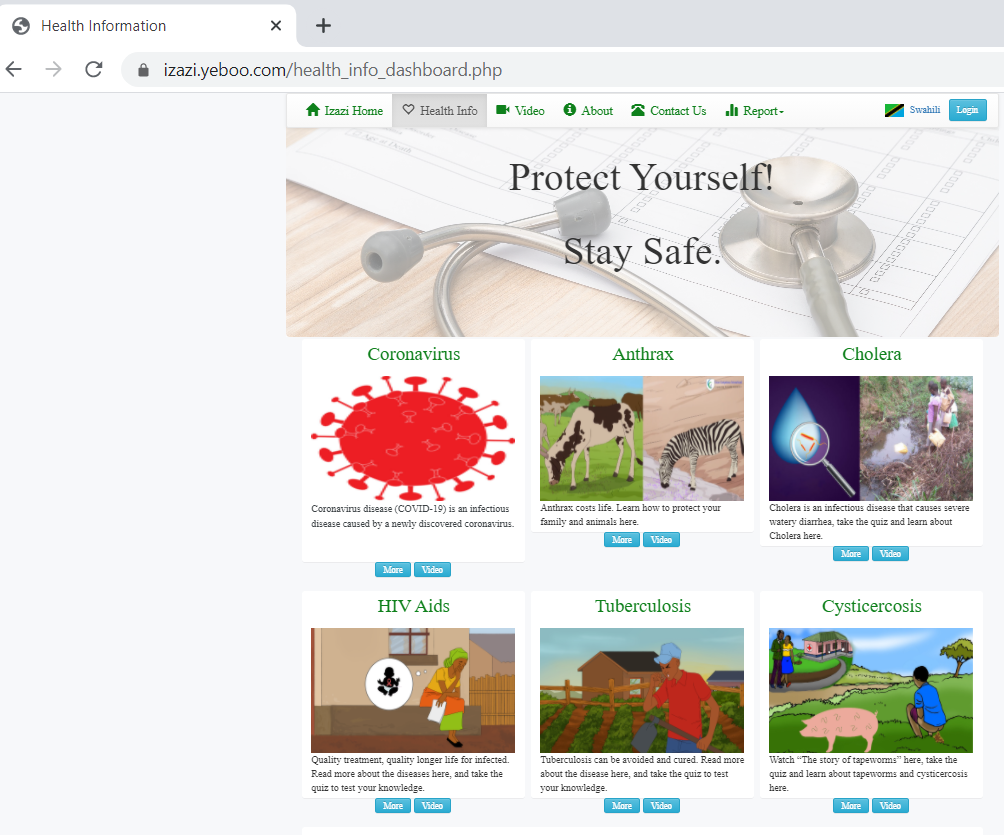

Supplement: Multimedia Appendix 4 [file resprot_v10i4e25128_app4.png]
