# Supplementary material for: Digital Health Intervention to Increase Health Knowledge Related to Diseases of High Public Health Concern in Iringa, Tanzania: Protocol for a Mixed Methods Study
Source: JMIR Res Protoc. 2021 Apr 22;10(4):e25128. doi: 10.2196/25128 (PMC8103301; doi:10.2196/25128)
Supplement: Multimedia Appendix 5 [file resprot_v10i4e25128_app5.docx]

Multimedia appendix 5 - Sampling plan all villages and sub-villages

| Sampling plan, all villages and sub-villages | | | | | | |
| --- | --- | --- | --- | --- | --- | --- |
| Village | Sub-village | Total no of HHs, April, 2019 | No of eligible HHs, April / May, 2019 | | No of randomly selected HHs | Proportion |
| Migoli | Migoli | 229 | | 91 | 51 | 0.169 |
|  | Mbuyuni | 380 | | 140 | 78 | 0.260 |
|  | Nyegele | 72 | | 26 | 14 | 0.048 |
|  | Nyerere | 148 | | 54 | 30 | 0.100 |
|  | **TOTAL** | **829** | | **311** | **173** | **0.577** |
| Izazi | Ihanyi | 91 | | 44 | 24 | 0.082 |
|  | Checkechea | 53 | | 46 | 26 | 0.085 |
|  | Sokoni | 79 | | 37 | 21 | 0.069 |
|  | Madukani | 45 | | 22 | 12 | 0.041 |
|  | Kiwanjani | 92 | | 42 | 23 | 0.078 |
|  | Barabarani | 72 | | 37 | 21 | 0.069 |
|  | **TOTAL** | **432** | | **228** | **127** | **0.423** |
| Intervention group | SUM HHs |  | | 539 |  |  |
| Intervention group | No of HHs needed |  | | 300 |  | 1.000 |
| Intervention group | No of HHs, Migoli |  | |  | 173 | 0.577 |
| Intervention group | No of HHs, Izazi |  | |  | 127 | 0.423 |
|  |  |  | |  |  |  |
| Kimande | Mji mwema | 171 | | 57 | 38 | 0.126 |
|  | Kikuluwe | 135 | | 56 | 37 | 0.124 |
|  | Kimande | 106 | | 31 | 20 | 0.069 |
|  | Igundambwanyi | 37 | | 21 | 14 | 0.046 |
|  | Mwatenga | 194 | | 63 | 42 | 0.139 |
|  | **TOTAL** | **643** | | **228** | **151** | **0.504** |
| Idodi | Mji mwema A | 83 | | 29 | 19 | 0.064 |
|  | Mji mwema B | 176 | | 53 | 35 | 0.117 |
|  | Msimbi | 97 | | 48 | 32 | 0.106 |
|  | Ilamba | 82 | | 25 | 17 | 0.055 |
|  | Mbuyuni A | 86 | | 30 | 20 | 0.066 |
|  | Mbuyuni B | 83 | | 39 | 26 | 0.086 |
|  | **TOTAL** | **524** | | **224** | **149** | **0.496** |
| Control group | SUM HHs |  | | 452 |  |  |
| Control group | No of HHs needed |  | | 300 |  | 1.000 |
| Control group | No of HHs, Kimande |  | |  | 151 | 0.504 |
| Control group | No of HHs, Idodi |  | |  | 149 | 0.496 |
