# Supplementary material for: Digital Health Intervention to Increase Health Knowledge Related to Diseases of High Public Health Concern in Iringa, Tanzania: Protocol for a Mixed Methods Study
Source: JMIR Res Protoc. 2021 Apr 22;10(4):e25128. doi: 10.2196/25128 (PMC8103301; doi:10.2196/25128)
Supplement: Multimedia Appendix 6 [file resprot_v10i4e25128_app6.pdf]

Baseline and immediate after, intervention and control group, Iringa April 1. - May 1.

DOI

____

Sex

- ○ Female
- ○ Male

Age

____

Village

- ○ Izazi
- ○ Migoli
- ○ Kimande
- ○ Idodi

Is the person willing to participate, and has the person signed the consent form?

- ● Yes
- ○ No

Why is the person not participating?

- ○ Away from the house for more than two days
- ○ Declining
- ○ No eligible household members
- ○ Has shifted to another place

What is your highest level of education

- ○ No education
- ○ Primary School
- ○ Secondary School
- ○ Post-secondary education

Occupation

- ○ Student
- ○ Public servant
- ○ Farmer
- ○ Fisherman
- ○ Business
- ○ Other

Other, spelace specify

____

Religion

- ○ Christian
- ○ Muslim
- ○ Other

Now, 14 HIV questions will follow

- ◉ OK

QH1: Have you ever heard of HIV and / or AIDS? (Check one box)

- ○ Yes
- ○ No
- ○ I don't know

QH2: How do you think a person can get HIV? (Check all boxes that apply)

- ☐ From mosquito bites
- ☐ From witchcraft
- ☐ From sharing food with HIV infected person
- ☐ By having unprotected sex with a person infected with HIV
- ☐ Through blood transfusion from a person infected with HIV
- ☐ Sharing sharp instruments
- ☐ Mother to child transmission
- ☐ I don't know

QH3: Do you know if a HIV infected pregnant woman can transmit HIV to her baby during pregnancy,delivery and breast feeding?

- ○ Yes
- ○ No
- ○ I don't know

QH4: Do you know if a HIV infected pregnant woman on ART can have a healthy baby?

- ◯ Yes
- ◯ No
- ◯ I don't know

QH5: Which group do you think is most vulnerable to HIV infection? (Check all boxes that apply)

- ☐ Girls
- ☐ Women
- ☐ Boys
- ☐ Men
- ☐ They are equally vulnerable
- ☐ I don't know

QH6: Do you think a healthy looking person can be infected with HIV? (Check one box)

- ◯ Yes
- ◯ No
- ◯ I don't know

QH7: How do you think a person knows that he or she is infected with HIV? (Check all boxes that apply)

- ☐ If the parents are HIV-positive
- ☐ If the results from a rapid test / blood sample is positive
- ☐ If the person has flu-like symptoms
- ☐ If the sexual partner is diagnosed with HIV infection
- ☐ I don't know

QH8: Does effective treatment of HIV infection by using antiretroviral therapy (ART)), reduces the risk of HIV transmission (Check one box)

- ◯ Yes
- ◯ No
- ◯ I don't know

QH9: What kind of treatment do you think is the most effective for HIV? (Check one box)

- ◯ Herbal treatment
- ◯ Antiretroviral therapy (ART)
- ◯ Witchcraft
- ◯ Prayers
- ◯ I don't know

QH10: Do you think there is a cure for HIV/AIDS? (Check one box)

- ◯ Yes
- ◯ No
- ◯ I don't know

QH11: What do you think is the most important an HIV-positive person on treatment can do for her / his own health? (Check all boxes that apply)

- ☐ Taking the medication prescribed by a medical doctor, every day at the same time and adhere to the doctor/nurse advice
- ☐ Practicing sexual abstinence
- ☐ Using a condom
- ☐ Taking some medication in periods
- ☐ I don't know

QH12: How do you think a person can prevent getting HIV? (Check all boxes that apply)

- ☐ Practicing sexual abstinence
- ☐ Using a condom
- ☐ Never sharing sharp objects(needles,razor blades,...)
- ☐ Never sharing food or drinks
- ☐ Never buying food from a HIV-infected person
- ☐ Never having casual contact with a HIV-positive person
- ☐ Avoiding unsafe blood transfusion
- ☐ I don't know

QH13: How do you think an HIV-infected person can prevent the spread of HIV? (Check all boxes that apply)

- ☐ Using a condom
- ☐ Practicing sexual abstinence
- ☐ Never sharing sharp objects(needles,razor blades,...)
- ☐ Avoiding unsafe blood transfusion
- ☐ Using HIV-medication as prevention, by reducing the amount of virus in the body
- ☐ Staying away from other people
- ☐ I don't know

QH14: According to your opinion, what are the major sources of information about HIV/AIDS? (Check all boxes that apply)

- ☐ Health workers / counsellors
- ☐ School
- ☐ Mass media (TV, radio, newspaper,...)
- ☐ Health facilities (hospital, dispensary,...)
- ☐ Posters and brochures
- ☐ Others..... (specify)
- ☐ Friends and family (Peer groups)
- ☐ Religious groups
- ☐ Other sources
- ☐ I don't know

Now, 10 TB questions will follow

- ○ OK

QT1: Have you ever heard of Tuberculosis (TB)? (Check one box)

- ○ Yes
- ○ No
- ○ I don't know

QT2: In which ways can TB be spread from one person to another? (Check all boxes that apply)

- ☐ Coughing
- ☐ Speaking
- ☐ Sneezing
- ☐ Spitting
- ☐ Laughing
- ☐ Singing
- ☐ I don't know

QT3: Do you think TB can also spread through the following ways? (List options) (Check all boxes that apply)

- ☐ Skin to skin contact, such as shaking hands
- ☐ In pregnancy from mother to child
- ☐ Mosquito bite
- ☐ None of the above
- ☐ I don't know

QT4: What are the symptoms of TB? (Check all boxes that apply)

- ☐ Coughing that lasts two or more weeks
- ☐ Coughing up blood
- ☐ Chest pain
- ☐ Pain with breathing or coughing
- ☐ Unintentional weight loss
- ☐ Fatigue
- ☐ Fever
- ☐ Night sweating
- ☐ Chills
- ☐ Loss of appetite
- ☐ I don't know

QT5: How do you think TB diagnosis is made? (Check all boxes that apply)

- ☐ Sputum examination
- ☐ Chest x-ray
- ☐ I don't know

QT6: What would you do if you had a fever, unexplained weight loss, drenching night sweats and a persistent cough? (Check all boxes that apply)

- ☐ See a traditional healer for herbal treatment
- ☐ See a health worker to check for TB and start a TB-treatment if positive
- ☐ Will do nothing but wait, to see if this passes
- ☐ I don't know

QT7: What do you think is the most important a person with TB disease can do for her / his own health? (Check all boxes that apply)

- ☐ Taking the medication prescribed by a medical doctor, every day at the same time and adhere to the doctor/nurse advice
- ☐ Staying away from people
- ☐ Taking some medication in periods
- ☐ I don't know

QT8: TB can be cured, even in people living with HIV, with a combination of ant-TB over a period of 6 months? (Check one box)

- ○ Yes
- ○ No
- ○ I don't know

QT9: What do you think a person with TB can do, in order to reduce transmission? (Check all boxes that apply)

- ☐ Cover his/her mouth and nose with the paper/piece of cloth when coughing
- ☐ Cough in a paper towel and then toss it in the pit toilet or burry it
- ☐ Cough or sneeze into the upper sleeve or elbow
- ☐ I don't know

QT10 How can TB be prevented in the general population?

- ☐ Adequate ventilated houses
- ☐ Always cover your mouth and nose during coughing and sneeze
- ☐ Avoid overcrowding
- ☐ BCG Vaccination in children
- ☐ Early TB detection and treatment
- ☐ TB preventive therapy in people living with HIV
- ☐ I don't know

Now, 17 cysticercosis questions will follow

- ○ OK

QC1: Have you ever heard of cysticercosis? (Check one box)

- ○ Yes
- ○ No
- ○ I don't know

QC2: Have you ever heard of pork tapeworm? (Check one box)

- ○ Yes
- ○ No
- ○ I don't know

QC3: Do you know how pork meat with cysticercosis looks like? (Check one box)

- ○ Yes
- ○ No
- ○ I don't know

QC4: Do you keep pigs at home? (Check one box)

- ○ Yes
- ○ No

QC5: Do you think a human being can also be infected with cysticercosis? (Check one box)

- ◯ Yes
- ◯ No
- ◯ I don't know

QC6: If yes, how can a person possibly acquire cysticercosis? (Check all boxes that apply)

- ☐ By eating raw or undercooked pork from infected pig
- ☐ By eating foods or drinking water contaminated with human faeces
- ☐ Insufficient personal hygiene, e.g. washing hands
- ☐ I don't know

QC7: Do you know how a person can acquire pork tapeworm infection? (Check all boxes that apply)

- ☐ By consuming raw or undercooked pork
- ☐ By consuming any raw or undercooked meat
- ☐ I don't know

QC8: How can one prevent a pig from cysticercosis? (Check one box)

- ◯ Cannot be prevented
- ◯ Use traditional medicine
- ◯ Prevent pigs access to human faeces
- ◯ I don't know

QC9: What are the signs of tapeworm infection in human? (Check all boxes that apply)

- ☐ Expulsion of tapeworm segment(s) in faeces
- ☐ Stomach ache, urge to vomit, weight loss, going to the toilet less often than usual and anaemia
- ☐ I don't know

QC10: In human, where do you think cysticercosis can be located? (Check all boxes that apply)

- ☐ In the muscles
- ☐ Under the skin
- ☐ In the brain
- ☐ In the eyes
- ☐ I don't know

QC11: What are the most important symptoms / signs of cysticercosis in the brain? (Check all boxes that apply)

- ☐ Epilepsy
- ☐ Severe progressive headache
- ☐ Fever
- ☐ Stroke
- ☐ I don't know

QC12: What are the symptoms of epilepsy in human? (Check all boxes that apply)

- ☐ Abdominal pain, vomiting and diarrhoea
- ☐ Fever
- ☐ Loss of consciousness and shaking of arms and legs
- ☐ I don't know

QC13: Do you know how a person with tapeworm infection and/ or cysticercosis can be treated? (Check all boxes that apply)

- ☐ Using traditional medicine
- ☐ Using modern medicine (go to hospital)
- ☐ I don't know

QC14: Which way of keeping pigs do you think is the best? (Check all boxes that apply)

- ☐ Free ranging pigs all the time
- ☐ Tethering the pigs all the time
- ☐ Housing the pigs all the time
- ☐ Housing the pigs at night and letting them to roam during the day
- ☐ I don't know

QC15: Do you know how a person can avoid tapeworm infection? (Check all boxes that apply)

- ☐ Eating meat only inspected by a government/private meat inspector
- ☐ Avoiding eating raw or undercooked meat
- ☐ Washing hands frequently, after toilet, and before preparing and eating food
- ☐ I don't know

QC16: What would you do if you encountered cysticercosis-infected meat? (Check one box)

- ○ Burn it completely
- ○ Cook meat thoroughly, and then consume it
- ○ I don't know

QC17: How can you avoid pigs from eating human faeces? (Check one box)

- ○ Tethering the pigs all the time
- ○ Housing the pigs all the time
- ○ Build an enclosed latrine, and use it
- ○ I don't know

Now, it is time to show the HIV video, before you ask the following 14 HIV questions again

- ○ OK

QH1: Have you ever heard of HIV and / or AIDS? (Check one box)

- ○ Yes
- ○ No
- ○ I don't know

QH2: How do you think a person can get HIV? (Check all boxes that apply)

- ☐ From mosquito bites
- ☐ From witchcraft
- ☐ From sharing food with HIV infected person
- ☐ By having unprotected sex with a person infected with HIV
- ☐ Through blood transfusion from a person infected with HIV
- ☐ Sharing sharp instruments
- ☐ Mother to child transmission
- ☐ I don't know

QH3: Do you know if a HIV infected pregnant woman can transmit HIV to her baby during pregnancy,delivery and breast feeding?

- ○ Yes
- ○ No
- ○ I don't know

QH4: Do you know if a HIV infected pregnant woman on ART can have a healthy baby?

- ○ Yes
- ○ No
- ○ I don't know

QH5: Which group do you think is most vulnerable to HIV infection? (Check all boxes that apply)

- ☐ Girls
- ☐ Women
- ☐ Boys
- ☐ Men
- ☐ They are equally vulnerable
- ☐ I don't know

QH6: Do you think a healthy looking person can be infected with HIV? (Check one box)

- ○ Yes
- ○ No
- ○ I don't know

QH7: How do you think a person knows that he or she is infected with HIV? (Check all boxes that apply)

- [ ] If the parents are HIV-positive
- [ ] If the results from a rapid test / blood sample is positive
- [ ] If the person has flu-like symptoms
- [ ] If the sexual partner is diagnosed with HIV infection
- [ ] I don't know

QH8: Does effective treatment of HIV infection by using antiretroviral therapy (ART)), reduces the risk of HIV transmission (Check one box)

- ○ Yes
- ○ No
- ○ I don't know

QH9: What kind of treatment do you think is the most effective for HIV? (Check one box)

- ○ Herbal treatment
- ○ Antiretroviral therapy (ART)
- ○ Witchcraft
- ○ Prayers
- ○ I don't know

QH10: Do you think there is a cure for HIV/AIDS? (Check one box)

- ○ Yes
- ○ No
- ○ I don't know

QH11: What do you think is the most important an HIV-positive person on treatment can do for her / his own health? (Check all boxes that apply)

- [ ] Taking the medication prescribed by a medical doctor, every day at the same time and adhere to the doctor/nurse advice
- [ ] Practicing sexual abstinence
- [ ] Using a condom
- [ ] Taking some medication in periods
- [ ] I don't know

QH12: How do you think a person can prevent getting HIV? (Check all boxes that apply)

- ☐ Practicing sexual abstinence
- ☐ Using a condom
- ☐ Never sharing sharp objects(needles,razor blades,...)
- ☐ Never sharing food or drinks
- ☐ Never buying food from a HIV-infected person
- ☐ Never having casual contact with a HIV-positive person
- ☐ Avoiding unsafe blood transfusion
- ☐ I don't know

QH13: How do you think an HIV-infected person can prevent the spread of HIV? (Check all boxes that apply)

- ☐ Using a condom
- ☐ Practicing sexual abstinence
- ☐ Never sharing sharp objects(needles,razor blades,...)
- ☐ Avoiding unsafe blood transfusion
- ☐ Using HIV-medication as prevention, by reducing the amount of virus in the body
- ☐ Staying away from other people
- ☐ I don't know

QH14: According to your opinion, what are the major sources of information about HIV/AIDS? (Check all boxes that apply)

- ☐ Health workers / counsellors
- ☐ School
- ☐ Mass media (TV, radio, newspaper,...)
- ☐ Health facilities (hospital, dispensary,...)
- ☐ Posters and brochures
- ☐ Others..... (specify)
- ☐ Friends and family (Peer groups)
- ☐ Religious groups
- ☐ Other sources
- ☐ I don't know

Now, it is time to show the TB-video before you ask the following 10 TB questions again

- ◯ OK

QT1: Have you ever heard of Tuberculosis (TB)? (Check one box)

- ◯ Yes
- ◯ No
- ◯ I don't know

QT2: In which ways can TB be spread from one person to another? (Check all boxes that apply)

- [ ] Coughing
- [ ] Speaking
- [ ] Sneezing
- [ ] Spitting
- [ ] Laughing
- [ ] Singing
- [ ] I don't know

QT3: Do you think TB can also spread through the following ways?... (Check all boxes that apply)

- [ ] Skin to skin contact, such as shaking hands
- [ ] In pregnancy from mother to child
- [ ] Mosquito bite
- [ ] None of the above
- [ ] I don't know

QT4: What are the symptoms of TB? (Check all boxes that apply)

- [ ] Coughing that lasts two or more weeks
- [ ] Coughing up blood
- [ ] Chest pain
- [ ] Pain with breathing or coughing
- [ ] Unintentional weight loss
- [ ] Fatigue
- [ ] Fever
- [ ] Night sweating
- [ ] Chills
- [ ] Loss of appetite
- [ ] I don't know

QT5: How do you think TB diagnosis is made? (Check all boxes that apply)

- [ ] Sputum examination
- [ ] Chest x-ray
- [ ] I don't know

QT6: What would you do if you had a fever, unexplained weight loss, drenching night sweats and a persistent cough? (Check all boxes that apply)

- ☐ See a traditional healer for herbal treatment
- ☐ See a health worker to check for TB and start a TB-treatment if positive
- ☐ Will do nothing but wait, to see if this passes
- ☐ I don't know

QT7: What do you think is the most important a person with TB disease can do for her / his own health? (Check all boxes that apply)

- ☐ Taking the medication prescribed by a medical doctor, every day at the same time and adhere to the doctor/nurse advice
- ☐ Staying away from people
- ☐ Taking some medication in periods
- ☐ I don't know

QT8: TB can be cured, even in people living with HIV, with a combination of ant-TB over a period of 6 months? (Check one box)

- ○ Yes
- ○ No
- ○ I don't know

QT9: What do you think a person with TB can do, in order to reduce transmission? (Check all boxes that apply)

- ☐ Cover his/her mouth and nose with the paper/piece of cloth when coughing
- ☐ Cough in a paper towel and then toss it in the pit toilet or burry it
- ☐ Cough or sneeze into the upper sleeve or elbow
- ☐ I don't know

QT10 How can TB be prevented in the general population?

- ☐ Adequate ventilated houses
- ☐ Always cover your mouth and nose during coughing and sneeze
- ☐ Avoid overcrowding
- ☐ BCG Vaccination in children
- ☐ Early TB detection and treatment
- ☐ TB preventive therapy in people living with HIV
- ☐ I don't know

Now, it is time to show the cysti-video before you ask the 17 cysti questions again

- ○ OK

QC1: Have you ever heard of cysticercosis? (Check one box)

- ○ Yes
- ○ No
- ○ I don't know

QC2: Have you ever heard of pork tapeworm? (Check one box)

- ○ Yes
- ○ No
- ○ I don't know

QC3: Do you know how pork meat with cysticercosis looks like? (Check one box)

- ○ Yes
- ○ No
- ○ I don't know

QC4: Do you keep pigs at home? (Check one box)

- ○ Yes
- ○ No

QC5: Do you think a human being can also be infected with cysticercosis? (Check one box)

- ○ Yes
- ○ No
- ○ I don't know

QC6: If yes, how can a person possibly acquire cysticercosis? (Check all boxes that apply)

- ☐ By eating raw or undercooked pork from infected pig
- ☐ By eating foods or drinking water contaminated with human faeces
- ☐ Insufficient personal hygiene, e.g. washing hands
- ☐ I don't know

QC7: Do you know how a person can acquire pork tapeworm infection? (Check all boxes that apply)

- ☐ By consuming raw or undercooked pork
- ☐ By consuming any raw or undercooked meat
- ☐ I don't know

QC8: How can one prevent a pig from cysticercosis? (Check one box)

- ○ Cannot be prevented
- ○ Use traditional medicine
- ○ Prevent pigs access to human faeces
- ○ I don't know

QC9: What are the signs of tapeworm infection in human? (Check all boxes that apply)

- [ ] Expulsion of tapeworm segment(s) in faeces
- [ ] Stomach ache, urge to vomit, weight loss, going to the toilet less often than usual and anaemia
- [ ] I don't know

QC10: In human, where do you think cysticercosis can be located? (Check all boxes that apply)

- [ ] In the muscles
- [ ] Under the skin
- [ ] In the brain
- [ ] In the eyes
- [ ] I don't know

QC11: What are the most important symptoms / signs of cysticercosis in the brain? (Check all boxes that apply)

- [ ] Epilepsy
- [ ] Severe progressive headache
- [ ] Fever
- [ ] Stroke
- [ ] I don't know

QC12: What are the symptoms of epilepsy in human? (Check all boxes that apply)

- [ ] Abdominal pain, vomiting and diarrhoea
- [ ] Fever
- [ ] Loss of consciousness and shaking of arms and legs
- [ ] I don't know

QC13: Do you know how a person with tapeworm infection and/ or cysticercosis can be treated? (Check all boxes that apply)

- [ ] Using traditional medicine
- [ ] Using modern medicine (go to hospital)
- [ ] I don't know

QC14: What way of keeping pigs do you think is the best? (Check all boxes that apply)

- [ ] Free ranging pigs all the time
- [ ] Tethering the pigs all the time
- [ ] Housing the pigs all the time
- [ ] Housing the pigs at night and letting them to roam during the day
- [ ] I don't know

QC15: Do you know how a person can avoid tapeworm infection? (Check all boxes that apply)

- Eating meat only inspected by a government/private meat inspector
- Avoiding eating raw or undercooked meat
- Washing hands frequently, after toilet, and before preparing and eating food
- I don't know

QC16: What would you do if you encountered cysticercosis-infected meat? (Check one box)

- Burn it completely
- Cook meat thoroughly, and then consume it
- I don't know

QC17: How can you avoid pigs from eating human faeces? (Check one box)

- Tethering the pigs all the time
- Housing the pigs all the time
- Build an enclosed latrine, and use it
- I don't know

We are almost there, now we would like to ask 5 questions on health promotion / information, and 5 questions on digital literacy

- OK

QHL1: Do you know how to read a leaflet with health information?

- Yes
- No

QHL2: How easy or hard is it to find good quality health information where you live?

- Very easy
- Easy
- Medium, not hard, not easy
- Hard
- Very hard

QHL3: Do you feel that you understand the health information you get well enough to know what to do?

- Yes
- No
- I don't know

QHL4: In your hometown, do you find good information on HIV / AIDS?

- Yes
- No
- I don't know

QHL5: In your hometown, do you find good information on Tuberculosis?

- ◯ Yes
- ◯ No
- ◯ I don't know

QHL6: In your hometown, do you find good information on cysticercosis / tapeworm?

- ◯ Yes
- ◯ No
- ◯ I don't know

QD1: How often on average did you use the internet in the last 3 months? * (Check one box)

- ◯ Every day or almost every day
- ◯ At least once a week (but not every day)
- ◯ Less than once a week
- ◯ None

QD2: On which of the following devices did you use the internet in the last 3 months? (Check all boxes that apply)

- ☐ Desktop computer
- ☐ Laptop
- ☐ Tablet
- ☐ Mobile phone or smart phone
- ☐ Other mobile devices (e.g. media or games player, e-book reader, etc.)
- ☐ None

QD3: For which of the following activities did you use the internet in the last 3 months? (Check all boxes that apply)

- ☐ Sending / receiving e-mails
- ☐ Telephoning over the internet / video calls (via webcam) over the internet (using applications, e.g. Skype)
- ☐ Participating in social networks (creating user profile, posting messages or other contributions to Facebook, twitter, Instagram, Snapchat, etc.)
- ☐ Listening to music (e.g. web radio, music streaming)
- ☐ Watching Video on Demand from commercial services
- ☐ Watching video content from sharing services
- ☐ Playing or downloading games
- ☐ eHealth
- ☐ Seeking health-related information (e.g. injuries, diseases, nutrition, improving health, etc.)
- ☐ Making an appointment with a practitioner via a website or apps (e.g. of a hospital or a health care centre)
- ☐ None of the above

QD4: Which internet service have you used the most in the past 3 months? (Check all boxes that apply)

- ☐ Search engine
- ☐ Browser (google, yahoo, opera, etc.)
- ☐ Email service
- ☐ Forums – interaction on forums, online discussion
- ☐ Online encyclopedias
- ☐ Other
- ☐ None of the above

QD5: Have you carried out any of the following learning activities to improve your skills relating to the use of computers, software or applications in the last 12 months? (Check all boxes that apply)

- ☐ Free online training or self-study
- ☐ Training paid by yourself
- ☐ Free training provided by public programs or organizations (other than your employer)
- ☐ Training paid or provided by your employer
- ☐ On-the-job training (e.g. co-workers, supervisors)
- ☐ None of the above
