# Supplementary material for: Digital Health Intervention to Increase Health Knowledge Related to Diseases of High Public Health Concern in Iringa, Tanzania: Protocol for a Mixed Methods Study
Source: JMIR Res Protoc. 2021 Apr 22;10(4):e25128. doi: 10.2196/25128 (PMC8103301; doi:10.2196/25128)
Supplement: Multimedia Appendix 7 [file resprot_v10i4e25128_app7.docx]

Multimedia appendix 7 - Interview guide semi-structured interviews (ENG)

1. Do you know how to read a health information leaflet? YES/ NO

2. Have you used the DigI hotspot in the village?

YES / NO

- if not – why not, please explain? If yes, please tell me about your experiences with it.

3. If yes in no 2: Have you looked at any of the free digital health messages?

YES/NO - (if yes, which ones?)

4. If yes in no 2: When did you use the hotspots, first and last time?

5. If yes in no 2: How often have you used it?

6. What do you think of the digital health message(s)?

Please explain - at least one negative thing and one positive thing.

7. What did you learn from them? (please tell me what you remember the most)

8. Have you explained the messages to any of your family members? (If yes, please tell me about your experiences with it.) (if not, why not?)

9. What can we improve? Please tell us what you think is not functioning optimally.

10. What is not user friendly? Please tell me if there are anything that you find difficult when using the hotspots.

11. Would you recommend it for someone else to use? Why? Why not?
